# Structural insights into the inhibition of *M. tuberculosis* Prolyl-tRNA synthetase by derivatives of 3-aminopyrazine-2-carboxamide

**DOI:** 10.1063/4.0001168

**Published:** 2025-10-27

**Authors:** Priyanka Gade, Karolina Michalska, Vinod Sukanth Kumar Pallabothula, Jan Zitko, Andrzej Joachimiak

**Affiliations:** 1 Center for Structural Biology of Infectious Diseases, Consortium for Advanced Science and Engineering, University of Chicago, Chicago, Illinois, USA; 2 Structural Biology Center, X-ray Science Division, Argonne National Laboratory, Lemont, Illinois, USA; 3 Faculty of Pharmacy in Hradec Králové, Charles University, Ak. Heyrovského 1203/8, 500 05 Hradec Králové, Czech Republic; 4 Department of Biochemistry and Molecular Biology, University of Chicago, Chicago, Illinois, USA

## Abstract

Tuberculosis (TB) is recognized as the second leading cause of death globally from a single infectious agent, following SARS-CoV-2 pneumonia. Aminoacyl-tRNA synthetases (aaRS) are essential enzymes responsible for attaching amino acids to their cognate tRNAs. These enzymes represent a promising set of targets for selective drug design due to the divergence between prokaryotic and eukaryotic aaRS. Recently, a new class of 3-aminopyrazine- 2-carboxamide derivatives has been identified as potent inhibitors of prolyl-tRNA synthetase (ProRS) from *Mycobacterium tuberculosis* (*Mtb)*. These compounds exhibit significant antimycobacterial activity against Multi Drug Resistant strains of *Mtb* and demonstrate cytotoxicity against HepG2 human hepatocellular carcinoma cells. In this study, we present the crystal structures of *Mtb*ProRS in complex with five distinct 3-aminopyrazine-2-carboxamide derivatives. Structural analysis reveals that these inhibitors compete with ATP for the binding site of *Mtb*ProRS. Importantly, a critical hydrogen bond between the Glu144 of *Mtb*ProRS and the amino group of the carboxamide is identified, providing insights into the selective inhibition of *Mtb*ProRS over human ProRS.

